# Association between serum uric acid and nonalcoholic fatty liver disease in community patients with type 2 diabetes mellitus

**DOI:** 10.7717/peerj.7563

**Published:** 2019-08-26

**Authors:** Linxin Xu, Ting Li, Jianhong Yin, Gang Lin, Yali Xu, Yi Ren, Yan Wang, Jing Yang, Liming Chen

**Affiliations:** 1 NHC Key Lab of Hormones and Development, Tianjin Key Lab of Metabolic Diseases, Metabolic Diseases Hospital & Institute of Endocrinology, Tianjin Medical University, Tianjin, China; 2 Department of Endocrinology, the First Hospital of Shanxi Medical University, Shanxi Medical University, Taiyuan, China; 3 Department of Endocrinology, Changzhi High-tech Development Zone Central Hospital, Shanxi, China

**Keywords:** Type 2 diabetes mellitus, Serum uric acid, Nonalcoholic fatty liver disease, Male sex

## Abstract

**Background:**

To investigate whether SUA is associated with NAFLD in men and women with T2DM.

**Methods:**

This cross-sectional study enrolled patients with T2DM at Shanxi High-Tech Development Zone Central Hospital (June 2011 to September 2017). Patients were stratified according to gender and presence/absence of NAFLD. Parameters associated with NAFLD were identified using multivariate stepwise linear regression and univariate/multivariate logistic regression.

**Results:**

Among 597 patients (325 males) enrolled, 352 had NAFLD. SUA was higher in the NAFLD group than in the non-NAFLD group for both men and women (*P* < 0.001). Multiple linear regression showed that body mass index (positively), triglycerides (positively) and estimated glomerular filtration rate (negatively) were independently related to SUA (*P* < 0.001). Univariate logistic regression revealed increased odds of NAFLD for SUA tertiles 2 (*P* = 0.022) and 3 (*P* = 0.001) in women and tertile 3 (*P* = 0.039) in men. After adjustment for multiple clinical parameters, SUA tertiles were significantly associated with NAFLD for tertile 3 in women (*P* = 0.014), although there were trends toward associations for tertile 2 in women (*P* = 0.074) and tertiles 2 and 3 in men (*P* = 0.085 and 0.054, respectively).

**Conclusion:**

SUA is not independently associated with NAFLD in men or women with T2DM after rigorous adjustment for other metabolic parameters.

## Introduction

Nonalcoholic fatty liver disease (NAFLD) is a major cause of chronic liver disease worldwide (Kitade et al., 2017). The global prevalence of NAFLD was recently estimated to be 24%, with the highest rates reported in South America and the Middle East followed by Asia, the USA and Europe (Younossi et al., 2018). NALFD has a wide spectrum of manifestations ranging from simple steatosis to non-alcoholic steatohepatitis, hepatic fibrosis, liver cirrhosis and even hepatocellular carcinoma (Kitade et al., 2017; Maiuolo et al., 2016; Mikolasevic et al., 2016; Neuschwander-Tetri, 2017). Previous research has indicated that NAFLD is associated with factors related to metabolic syndrome, including obesity, dyslipidemia, impaired insulin resistance and type-2 diabetes mellitus (T2DM) (Kitade et al., 2017; Maiuolo et al., 2016; Mikolasevic et al., 2016; Neuschwander-Tetri, 2017).

Serum uric acid (SUA) is an end product generated from the metabolism of purine nucleotides (Maiuolo et al., 2016). Previous investigations have reported positive associations of SUA with obesity (Duan et al., 2015), dyslipidemia (Wang et al., 2015), insulin resistance (De Miranda et al., 2015), prediabetes (Van der Schaft et al., 2017) and T2DM (Xue et al., 2015). Furthermore, SUA was found to be an independent predictor of T2DM, metabolic syndrome (or its components) and cardiovascular mortality (Choi et al., 2016; Fu et al., 2017; Lv et al., 2013; Wang et al., 2016; Xue et al., 2015; Zheng et al., 2017). This raises the possibility that elevated SUA may be involved in the development or/and progression of these disorders.

In recent years, several published studies have highlighted a relation between SUA and NAFLD (Darmawan, Hamijoyo & Hasan, 2017; Jaruvongvanich et al., 2017; Zhou, Wei & Fan, 2016). The level of SUA is elevated in people with NAFLD (Cai et al., 2014; Lin et al., 2015; Liu et al., 2016; Moon, 2013). Moreover, an increased SUA level was reported to be associated with a higher prevalence of NAFLD in the general population (Cai et al., 2014; Cai et al., 2013; Lee et al., 2010a; Lee et al., 2010b; Li et al., 2009; Shih et al., 2015; Sirota et al., 2013; Yamada et al., 2010). Longitudinal studies have shown that an increase in the level of SUA is associated with a higher risk of new-onset NAFLD (Bai et al., 2018; Lu et al., 2016). SUA concentration is also related to greater NAFLD severity (Liu et al., 2017; Petta et al., 2011; Sirota et al., 2013). Interestingly, gender differences in the relation between SUA and NAFLD have been reported, with some studies suggesting a stronger association in men (Fan et al., 2016; Yu et al., 2017) and others a stronger association in women (Wu et al., 2015).

A small number of previous studies have indicated that SUA level is independently associated with NAFLD in patients with prediabetes (Hossain et al., 2016) or T2DM (Fan et al., 2016; Li et al., 2015). Furthermore, urine uric acid excretion is also related to NAFLD in patients with T2DM (Zhao et al., 2016). Only one previous investigation has explored whether the relation between SUA and NAFLD differs between men and women with T2DM, with the results indicating an association only in male patients (Fan et al., 2016). Nevertheless, data are very limited regarding gender-specific associations between SUA and NAFLD in people with T2DM. We hypothesized that SUA would be independently associated with NAFLD in patients with T2DM and that this association would show differences between men and women. Therefore, we investigated whether SUA was independently associated with NAFLD in patients with T2DM, and we performed a stratified analysis based on gender.

## Patients and Methods

### Study design and participants

This cross-sectional study enrolled consecutive patients with T2DM seen at the Department of Endocrinology, Shanxi High-Tech Development Zone Central Hospital, Changzhi, China between June 2011 and September 2017. The inclusion criterion was a diagnosis of T2DM made according to the 1999 World Health Organization standards (Organization, 1999). The diagnosis of NAFLD was made if all three of the following criteria were met (Fan et al., 2011): (1) alcohol consumption <140 g/week for men and <70 g/week for women; (2) absence of any specific disease that might induce a fatty liver, such as viral hepatitis, drug-induced hepatitis, total parenteral nutrition, hepatolenticular degeneration or autoimmune liver disease; and (3) pathologic examinations of liver biopsies yielded results consistent with the diagnostic criteria for fatty liver. Since it was not possible to obtain liver tissue specimens from many patients, the following working definition of NAFLD was used when necessary: (1) the ultrasound imaging features of the liver met the diagnostic criteria for diffuse fatty liver that could not be explained by other causes; and/or (2) unexplained elevations in serum alanine aminotransferase (ALT), aspartate aminotransferase (AST) and/or gamma-glutamyl transferase (GGT) for at least 6 months in patients with components of metabolic syndrome.

All study participants were asked to complete a standardized questionnaire that collected information regarding past and present medical history, including any therapies received. The data from the questionnaire were used to apply the following exclusion criteria: alcohol intake >140 g/week for men and >70 g/week for women; history of viral hepatitis, autoimmune hepatitis or other chronic liver disease; history of heart failure; history of renal dysfunction; and taking medications that might affect SUA. The study was designed and performed in accordance with the Helsinki Declaration and was approved by the Ethics Committee of The First Hospital of Shanxi Medical University (approval number: 2018 [K002]). All study participants provided informed written consent.

### Collection of demographic and clinical data

The age and gender of each participant were recorded along with data for duration of T2DM (i.e., time since diagnosis), consumption of tobacco and alcohol, and use of statins and anti-hypertensive drugs. A suitably trained physician measured the height, weight, waist circumference, hip circumference, systolic blood pressure (SBP) and diastolic blood pressure (DBP) of each participant. The waist-to-hip ratio (WHR) was calculated as the ratio of the waist circumference (in cm) to the hip circumference (in cm), and body mass index (BMI) was calculated as weight (in kg) divided by height (in m) squared. An experienced nurse obtained venous blood samples for measurement of total cholesterol (TC), triglycerides (TG), low-density lipoprotein cholesterol (LDL-C), high-density lipoprotein cholesterol (HDL-C), ALT, AST, GGT, serum creatinine (Scr), SUA, glycated hemoglobin (HbA1c), fasting plasma glucose (FPG), plasma glucose 2 h after a 75 g oral glucose load (2hPG), fasting insulin (FINS) and fasting C-peptide (FC-P). Homeostasis model assessment of insulin resistance (HOMA-IR) was calculated as FINS (mIU/L) × FPG (mmol/L) / 22.5. Estimated glomerular filtration rate (eGFR) was calculated using the Chronic Kidney Disease Epidemiology Collaboration (CKD-EPI) formula (Levey et al., 2009): for females, eGFR (mL/min per 1.73 m^2^) =144 × [Scr (mg/dL)/0.7]^−0.329^ × [0.993]^age^ if Scr ≤0.7 mg/dL and 144 × [Scr (mg/dL) / 0.7]^−1.209^ × [0.993]^age^ if Scr >0.7 mg/dL; and for males, eGFR (mL/min per 1.73 m^2^) =141 × [Scr (mg/dL)/0.9]^−0.411^ × [0.993]^age^ if Scr ≤0.9 mg/dL and 141 × [Scr (mg/dL) / 0.7]^−1.209^ × [0.993]^age^ if Scr >0.9 mg/dL.

### Statistical analysis

Data analyses were carried out using SPSS 13.0 (SPSS Inc., Chicago, IL, USA). Continuous variables are expressed as the mean ± standard deviation (SD) or median (interquartile range). Count data are presented as *n* (%). Non-normally distributed data were logarithmically transformed before analysis. Comparisons of continuous variables between two groups were made using Student’s *t*-test. Pearson correlation analysis and multivariate stepwise linear regression were used to analyze the associations of SUA with age, duration of T2DM, SBP, DBP, TC, TG, LDL-C, HDL-C, FPG, HbA1c, AST, ALT, HOMA-IR and eGFR. Univariate and multivariate logistic regression analyses were performed to identify factors independently associated with NAFLD in patients with T2DM. The multivariate logistic regression analysis used the ‘enter’ method (i.e., simultaneous entry of all factors into the logistic regression equation), and odds ratios (ORs) and 95% confidence intervals (95% CIs) were calculated. Three models were used for the multivariate logistic regression analyses: model 1 (adjusted for age, duration of T2DM, BMI, SBP, alcohol intake and eGFR), model 2 (adjusted for factors in model 1, TG, HbA1c, HOMA-IR, statin use, anti-hypertensive drug use and history of hyperuricemia) and model 3 (adjusted for factors in model 2, C-peptide, HDL-C and, for females only, ALT and AST). The above analyses were carried out stratified for gender. Gender-specific SUA tertiles were defined as follows: tertile 1, <280 µmol/L for all patients, <300 µmol/L for males and <260 µmol/L for females; tertile 2, 280–349 µmol/L for all patients, 300–361 µmol/L for males and 260–328 µmol/L for females; and tertile 3, >349 µmol/L for all patients, >360 µmol/L for males and >328 µmol/L for females. *P* < 0.05 was considered statistically significant.

## Results

### Demographic and clinical characteristics of the study participants

A total of 597 patients (325 males, 54.5%) with T2DM satisfied the inclusion and exclusion criteria. Among these 597 patients, 352 (59.0%) had NAFLD. The clinical characteristics of the patients stratified according to SUA tertile are shown in Table 1. The prevalence of NAFLD, BMI, waist circumference, WHR, DBP, TG, ALT, AST, GGT, HOMA-IR and Scr increased progressively with an increase of SUA level (*P* < 0.05). Hyperuricemia was observed in a small minority of patients categorized as SUA tertile 3 but was not observed in any patients classified as SUA tertile 1 or 2.

**Table 1:** Clinical characteristics of patients with type 2 diabetes in the community stratified according to serum uric acid tertile.

	All patients				Men				Women			
	SUA tertile 1	SUA tertile 2	SUA tertile 3	*P* for trend	SUA tertile 1	SUA tertile 2	SUA tertile 3	*P* for trend	SUA tertile 1	SUA tertile 2	SUA tertile 3	*P* for trend
n	201	195	201		108	109	108		91	92	89	
Age (years)	54 ± 12	54 ± 13	52 ± 14	0.067	52 ± 11.5	52 ± 13	49 ± 14	0.183	55 ± 13	57 ± 12	56 ± 15	0.785
T2DM duration (years)	8 (3, 13)	9 (4, 15)	7.0 (2.3, 13.0)	0.534	7 (3, 12)	8 (3, 14)	7 (1.3, 12)	0.690	10 (4, 15)	10 (5, 15)	10 (3.5, 15)	0.934
BMI (kg/m^2^)	24.8 ± 4.9	25.6 ± 3.6	26.9 ± 4.0	<0.001	24.9 ± 3.3	25.9 ± 3.4	26.8 ± 3.7	<0.001	24.4 ± 6.3	25.5 ± 3.3	27.0 ± 4.7	0.001
Waist circumference (cm)	89.7 ± 10.3	93.5 ± 10.0	96.1 ± 10.7	<0.001	92.1 ± 10.7	94.1 ± 9.8	97.4 ± 10.4	<0.001	86.8 ± 9.4	92.7 ± 9.3	94.9 ± 11.4	<0.001
WHR	0.9 ± 0.07	0.9 ± 0.07	0.94 ± 0.07	<0.001	0.91 ± 0.07	0.94 ± 0.06	0.95 ± 0.07	<0.001	0.89 ± 0.06	0.92 ± 0.08	0.93 ± 0.08	0.001
SBP (mmHg)	129 ± 18	132 ± 19	129 ± 17	0.812	128 ± 17	130 ± 18	128 ± 15	0.832	131 ± 19	134 ± 22	130 ± 16	0.888
DBP (mmHg)	78 ± 11	80 ± 10	81 ± 11	0.013	80 ± 12	81 ± 10	82 ± 11	0.240	76 ± 10	79 ± 11	78 ± 11	0.242
TC (mmol/L)	4.6 ± 0.9	4.8 ± 1.1	4.8 ± 1.3	0.114	4.6 ± 0.9	4.5 ± 1.0	4.8 ± 1.5	0.330	4.7 ± 1.0	4.8 ± 1.2	4.9 ± 1.1	0.100
TG (mmol/L)	1.4 (1.0, 1.9)	1.6 (1.1, 2.2)	1.9 (1.3, 3.0)	<0.001	1.3 (1.0, 1.9)	1.5 (1.1, 2.2)	1.8 (1.4, 3.1)	0.003	1.3 (1.0, 1.9)	1.6 (1.2, 2.3)	2.0 (1.5, 2.7)	<0.001
HDL-C (mmol/L)	1.0 ± 0.3	0.97 ± 0.24	0.95 ± 0.25	0.003	0.95 ± 0.21	0.93 ± 0.25	0.89 ± 0.24	0.049	1.09 ± 0.28	1.03 ± 0.27	1.00 ± 0.20	0.025
LDL-C (mmol/L)	2.8 ± 0.8	2.9 ± 0.9	2.8 ± 0.9	0.876	2.9 ± 0.8	2.7 ± 0.8	2.8 ± 0.8	0.145	2.8 ± 0.8	2.8 ± 0.9	2.9 ± 1.0	0.402
ALT (IU/L)	15 (12, 23)	16 (12, 25)	23 (14, 34)	<0.001	16 (13, 26)	17 (12, 27)	24 (16, 37)	0.001	14 (11, 22)	16 (11, 22)	20 (13, 33)	<0.001
AST (IU/L)	16 (14, 20)	17 (13, 23)	20 (15, 27)	<0.001	17 (13, 20)	17 (14, 22)	20 (15, 25)	0.117	16 (13, 20)	16 (14, 22)	20 (14, 32)	<0.001
GGT (IU/L)	20 (15, 29)	26 (17, 37)	30 (21, 50)	<0.001	22 (16, 35)	28 (20, 46)	37 (23, 58)	<0.001	17 (13, 23)	22 (15, 32)	27 (19, 39)	0.011
Scr (µmol/L)	53 (45, 63)	60 (51, 70)	64 (54, 74)	<0.001	66 (56, 72)	63 (57, 73)	67 (61, 76)	0.013	46 (42, 54)	50 (44, 59)	56 (46, 65)	<0.001
eGFR (mL/min/1.73 m^2^)	164.4 ± 43.2	151.5 ± 40.5	146.8 ± 42.6	<0.001	163.0 ± 45.0	155.7 ± 41.3	150.0 ± 39.6	0.022	166.4 ± 40.3	152.9 ± 44.0	136.2 ± 40.5	<0.001
HbA1c (%)	9.5 ± 2.4	9.1 ± 1.9	9.1 ± 2.0	0.030	9.8 ± 2.2	9.2 ± 2.0	9.1 ± 2.1	0.018	9.2 ± 2.5	9.3 ± 2.0	8.8 ± 1.8	0.242
FPG (mmol/L)	8.9 ± 3.3	8.5 ± 2.6	8.3 ± 2.6	0.037	9.3 ± 3.0	8.3 ± 2.5	8.4 ± 2.7	0.028	8.4 ± 3.1	8.9 ± 3.3	8.0 ± 2.3	0.411
2hPG (mmol/L)	13.5 ± 4.9	12.9 ± 4.2	12.7 ± 4.1	0.078	13.8 ± 4.8	12.8 ± 4.3	13.0 ± 4.5	0.215	13.4 ± 4.8	13.0 ± 4.2	12.2 ± 3.6	0.071
FINS (mIU/L)	4.1 ± 2.6	4.6 ± 2.8	5.0 ± 2.8	0.001	4.2 ± 2.5	4.4 ± 2.2	5.0 ± 2.5	0.012	3.7 ± 2.6	4.5 ± 2.9	5.4 ± 3.5	<0.001
HOMA-IR	1.5 ± 1.0	1.7 ± 1.04	1.7 ± 1.0	0.033	1.6 ± 1.0	1.6 ± 0.9	1.8 ± 0.9	0.237	1.3 ± 0.9	1.7 ± 1.2	1.8 ± 1.1	0.002
FC-P (nmol/L)	2.1 ± 1.2	2.1 ± 1.5	2.0 ± 1.1	0.264	2.2 ± 1.1	2.1 ± 1.5	2.0 ± 0.9	0.245	2.2 ± 1.3	1.9 ± 1.2	2.1 ± 1.5	0.772
Anti-hypertensive drug use	64 (31.8%)	79 (40.5%)	76 (37.8%)	0.185	31 (28.7%)	38 (34.9%)	36 (33.3%)	0.601	29 (31.9%)	40 (43.5%)	45 (50.6%)	0.037
Statin use	25 (12.4%)	31 (15.9%)	22 (10.9%)	0.326	8 (7.4%)	10 (9.2%)	11 (10.2%)	0.769	13 (14.3%)	17 (18.5%)	19 (21.3%)	0.463
Hyperuricemia	0 (0%)	0 (0%)	9 (4.5%)	<0.001	0 (0%)	0 (0%)	7 (6.5%)	<0.001	0 (0%)	0 (0%)	2 (2.2%)	0.102

**Notes.**

Data are presented as mean ± standard deviation, median (interquartile range) or *n* (%).

2hPG plasma glucose 2 h after a 75 g oral glucose load ALT alanine aminotransferase AST aspartate aminotransferase BMI body mass index DBP diastolic blood pressure eGFR estimated glomerular filtration rate FC-P fasting C-peptide FINS fasting insulin FPG fasting plasma glucose GGT gamma-glutamyl transferase HbA1c glycated hemoglobin HDL-C high-density lipoprotein cholesterol HOMA-IR homeostasis model assessment of insulin resistance LDL-C low-density lipoprotein cholesterol NAFLD nonalcoholic fatty liver disease SBP systolic blood pressure Scr serum creatinine; SUA: serum uric acid T2DM type 2 diabetes mellitus TC total cholesterol TG triglycerides WHR waist-to-hip ratio

**Table 2:** Comparison of the clinical characteristics of patients with T2DM in the community between those with NAFLD and those without NAFLD.

		**All patients**			**Men**			**Women**	
	**Non-NAFLD**	**NAFLD**	***P***	**Non-NAFLD**	**NAFLD**	***P***	**Non-NAFLD**	**NAFLD**	***P***
*n*	245	352		125	200		120	152	
Age (years)	56 ± 13	52 ± 13	<0.001	54.3 ± 11.9	49.2 ± 13.2	<0.001	57.3 ± 13.2	54.8 ± 12.9	0.122
T2DM duration (years)	10 (5, 16)	7 (2, 12)	<0.001	10 (4, 15)	5 (1, 10)	<0.001	11 (6, 17)	8 (3, 13)	0.001
BMI (kg/m^2^)	24.2 ± 4.5	26.8 ± 3.8	<0.001	24.1 ± 3.0	27.0 ± 3.4	<0.001	24.3 ± 5.7	26.7 ± 4.2	<0.001
Waist (cm)	88.8 ± 9.8	96.1 ± 10.2	<0.001	89.4 ± 9.7	97.7 ± 9.8	<0.001	88.2 ± 9.9	94.0 ± 10.5	<0.001
WHR	0.90 ± 0.07	0.94 ± 0.07	<0.001	0.91 ± 0.06	0.95 ± 0.07	<0.001	0.90 ± 0.08	0.92 ± 0.08	0.064
SBP (mmHg)	131 ± 19	130 ± 17	0.526	129.0 ± 16.8	128.8 ± 16.5	0.918	132.4 ± 21.3	130.9 ± 17.0	0.527
DBP (mmHg)	78 ± 11	80 ± 11	0.031	80.1 ± 10.6	81.7 ± 11.0	0.209	76.5 ± 10.3	78.6 ± 11.5	0.126
Alcohol drinker, *n* (%)	41 (16.7)	65 (18.5)	0.923	41 (32.8)	62 (31.0)	0.590	0 (0)	3 (1.97)	0.330
TC (mmol/L)	4.7 ± 1.2	4.8 ± 1.1	<0.010	4.5 ± 1.3	4.7 ± 1.1	0.019	4.8 ± 1.1	4.8 ± 1.0	0.745
TG (mmol/L)	1.2 (0.9, 1.6)	1.9 (1.4, 2.7)	<0.001	1.14 (0.86, 1.54)	1.84 (1.40, 2.92)	<0.001	1.37 (0.95, 1.84)	1.96 (1.36, 2.73)	<0.001
HDL-C (mmol/L)	1.1 ± 0.3	0.9 ± 0.2	<0.001	1.03 ± 0.29	0.87 ± 0.18	<0.001	1.12 ± 0.27	0.98 ± 0.22	<0.001
LDL-C (mmol/L)	2.8 ± 0.8	2.8 ± 0.8	0.968	2.75 ± 0.81	2.84 ± 0.77	0.308	2.89 ± 0.84	2.78 ± 0.93	0.322
ALT (IU/L)	14 (11, 19)	22 (14, 34)	<0.001	15 (12, 20)	24.0 (16.0, 37.0)	<0.001	14 (10, 18)	19.0 (12.0, 31.0)	<0.001
AST (IU/L)	16 (13, 20)	19 (15, 26)	<0.001	16.0 (13.0, 19.0)	19.0 (15.0, 25.0)	<0.001	16.0 (13.3, 21.0)	18.5 (14.0, 26.8)	0.023
GGT (IU/L)	19 (14, 26)	30 (20, 47)	<0.001	21.0 (16.0, 29.0)	35.0 (23.3, 54.0)	<0.001	17.0 (14.0, 24.0)	24.5 (18.0, 35.8)	<0.001
Scr (µmol/L)	60 (50, 70)	59 (49, 68)	0.260	67.0 (60.0, 74.0)	65.0 (57.0, 72.0)	0.139	52.0 (44.0, 59.0)	50.0 (45.0, 57.8)	0.270
SUA (µmol/L)	300 ± 86	338 ± 86	<0.001	316 ± 76	352 ± 88	<0.001	283 ± 93	321 ± 80	<0.001
eGFR (mL/min/1.73 m^2^)	149.4 ± 43.2	157.6 ± 42.1	0.021	150.1 ± 39.4	159.9 ± 43.6	0.021	148.7 ± 47.1	154.6 ± 40.0	0.274
HbA1c (%)	9.2 ± 2.4	9.3 ± 1.9	0.579	9.24 ± 2.35	9.42 ± 1.99	0.460	9.14 ± 2.41	9.12 ± 1.83	0.929
FPG (mmol/L)	8.3 ± 3.1	8.8 ± 2.7	0.151	8.39 ± 2.75	8.82 ± 2.79	0.176	8.30 ± 3.36	8.52 ± 2.67	0.547
2hPG (mmol/L)	12.6 ± 4.4	13.4 ± 4.4	0.032	12.34 ± 4.37	13.72 ± 4.58	0.007	12.81 ± 4.51	12.88 ± 4.11	0.892
FINS (mIU/L)	3.6 ± 2.4	5.2 ± 2.8	<0.001	3.57 ± 2.14	5.17 ± 2.41	<0.001	3.54 ± 2.63	5.33 ± 3.16	<0.001
HOMA-IR	1.2 ± 0.8	1.9 ± 1.0	<0.001	1.16 (0.73, 1.75)	1.75 (1.33, 2.44)	<0.001	1.0 (0.53, 1.65)	1.67 (1.10, 2.62)	<0.001
FC-P (nmol/L)	2.04 ± 1.15	2.11 ± 1.31	0.505	2.02 ± 1.03	2.14 ± 1.28	0.387	2.07 ± 1.27	2.08 ± 1.36	0.939
Anti-hypertensive drug use	89 (36.3%)	130 (36.9%)	0.880	42 (33.6%)	63 (31.5%)	0.694	47 (39.2%)	67 (44.1%)	0.415
Statin use	30 (12.2%)	48 (13.6%)	0.620	10 (8.0%)	19 (9.5%)	0.644	20 (16.7%)	29 (19.1%)	0.607
Hyperuricemia	1 (0.4%)	8 (2.3%)	0.089	0 (0%)	7 (3.5%)	0.046	1 (0.8%)	1 (0.7%)	1.000

**Notes.**

Data are presented as mean ± standard deviation, median (interquartile range) or *n* (%).

2hPG plasma glucose 2 h after a 75 g oral glucose load ALT alanine aminotransferase AST aspartate aminotransferase; BMI: body mass index DBP diastolic blood pressure eGFR estimated glomerular filtration rate FC-P fasting C-peptide FINS fasting insulin FPG fasting plasma glucose GGT gamma-glutamyl transferase HbA1c glycated hemoglobin HDL-C high-density lipoprotein cholesterol HOMA-IR homeostasis model assessment of insulin resistance LDL-C low-density lipoprotein cholesterol NAFLD nonalcoholic fatty liver disease SBP systolic blood pressure Scr serum creatinine SUA serum uric acid T2DM type 2 diabetes mellitus TC total cholesterol TG triglycerides WHR waist-to-hip ratio

**Table 3:** Pearson correlation analysis and multiple linear regression analysis of parameters associated with serum uric acid.

	Men				Women			
	Pearson correlation		Multiple linear regression		Pearson correlation		Multiple linear regression	
	*r*	*P*	Standardized *β*	*P*	*r*	*P*	Standardized *β*	*P*
Age	−0.072	0.198	–	–	0.020	0.739	–	–
Duration of T2DM	−0.020	0.715	–	–	0.099	0.104	–	–
BMI	0.301	<0.001	0.270	<0.001	0.229	<0.001	0.186	0.001
SBP	−0.012	0.830	–	–	0.003	0.956	–	–
DBP	0.105	0.058	–	–	0.049	0.421	–	–
TG	0.204	<0.001	0.174	0.001	0.292	<0.001	0.270	<0.001
TC	0.107	0.055	–	–	0.061	0.318	–	–
LDL-C	−0.007	0.906	–	–	−0.038	0.534	–	–
HDL-C	−0.097	0.082	–	–	−0.156	0.010	–	–
FPG	−0.069	0.216	–	–	−0.049	0.422	–	–
HbA1C	−0.105	0.060	–	–	−0.055	0.370	–	–
eGFR	−0.117	0.035	−0.140	0.008	−0.281	<0.001	−0.301	<0.001
HOMA-IR	0.135	0.015	–	–	0.128	0.034	–	–
AST	0.109	0.049			0.261	<0.001	0.362	0.001
ALT	0.226	<0.001			0.166	0.006	0.219	0.046

**Notes.**

ALT alanine aminotransferase AST aspartate aminotransferase BMI body mass index DBP diastolic blood pressure eGFR estimated glomerular filtration rate FPG fasting plasma glucose HbA1c glycated hemoglobin HDL-C high-density lipoprotein cholesterol HOMA-IR homeostasis model assessment of insulin resistance LDL-C low-density lipoprotein cholesterol SBP systolic blood pressure T2DM type 2 diabetes mellitus TC total cholesterol TG triglycerides.

### Comparison of the clinical characteristics between T2DM patients with and without NAFLD

The clinical characteristics of the 597 study participants, stratified according to gender and presence/absence of NAFLD, are presented in Table 2. When compared with patients without NAFLD, those with NAFLD were younger (*P* < 0.001) and had a shorter T2DM disease course (*P* < 0.001), higher BMI (*P* < 0.001), larger waist circumference (*P* < 0.001), greater WHR (*P* < 0.001), lower HDL-C (*P* < 0.001), and higher DBP (*P* = 0.031), TC (*P* < 0.010), TG (*P* < 0.001), 2hPG (*P* = 0.032), FINS (*P* < 0.001), HOMA-IR (*P* < 0.001), ALT (*P* < 0.001), GGT (*P* < 0.001), AST (*P* < 0.001) and eGFR (*P* = 0.021). In comparison to men without NAFLD, men with NAFLD were younger (*P* < 0.001) and had a shorter T2DM disease course (*P* < 0.001), higher BMI (*P* < 0.001), larger waist circumference (*P* < 0.001), greater WHR (*P* < 0.001), lower HDL-C (*P* < 0.001), and higher TC (*P* = 0.019), TG (*P* < 0.001), 2hPG (*P* = 0.007), FINS (*P* < 0.001), HOMA-IR (*P* < 0.001), ALT (*P* < 0.001), GGT (*P* < 0.001), AST (*P* < 0.001) and eGFR (*P* = 0.021). Compared to women without NAFLD, women with NAFLD had a shorter T2DM disease course (*P* = 0.001), higher BMI (*P* < 0.001), larger waist circumference (*P* < 0.001), lower HDL-C (*P* < 0.001), and higher TG (*P* < 0.001), FINS (*P* < 0.001), HOMA-IR (*P* < 0.001), ALT (*P* < 0.001), GGT (*P* < 0.001) and AST (*P* = 0.023). Notably, the SUA level was significantly higher in the NAFLD group than in the non-NAFLD group (338 ± 86 *vs* 300 ± 86 µmol/L, *P* < 0.001), and this was also the case for women (351.6 ± 88.1 *vs* 316.1 ± 76.4 µmol/L, *P* < 0.001) and men (321.1 ± 80.1 *vs* 283.1 ± 92.8 µmol/L, *P* < 0.001).

**Figure 1:**
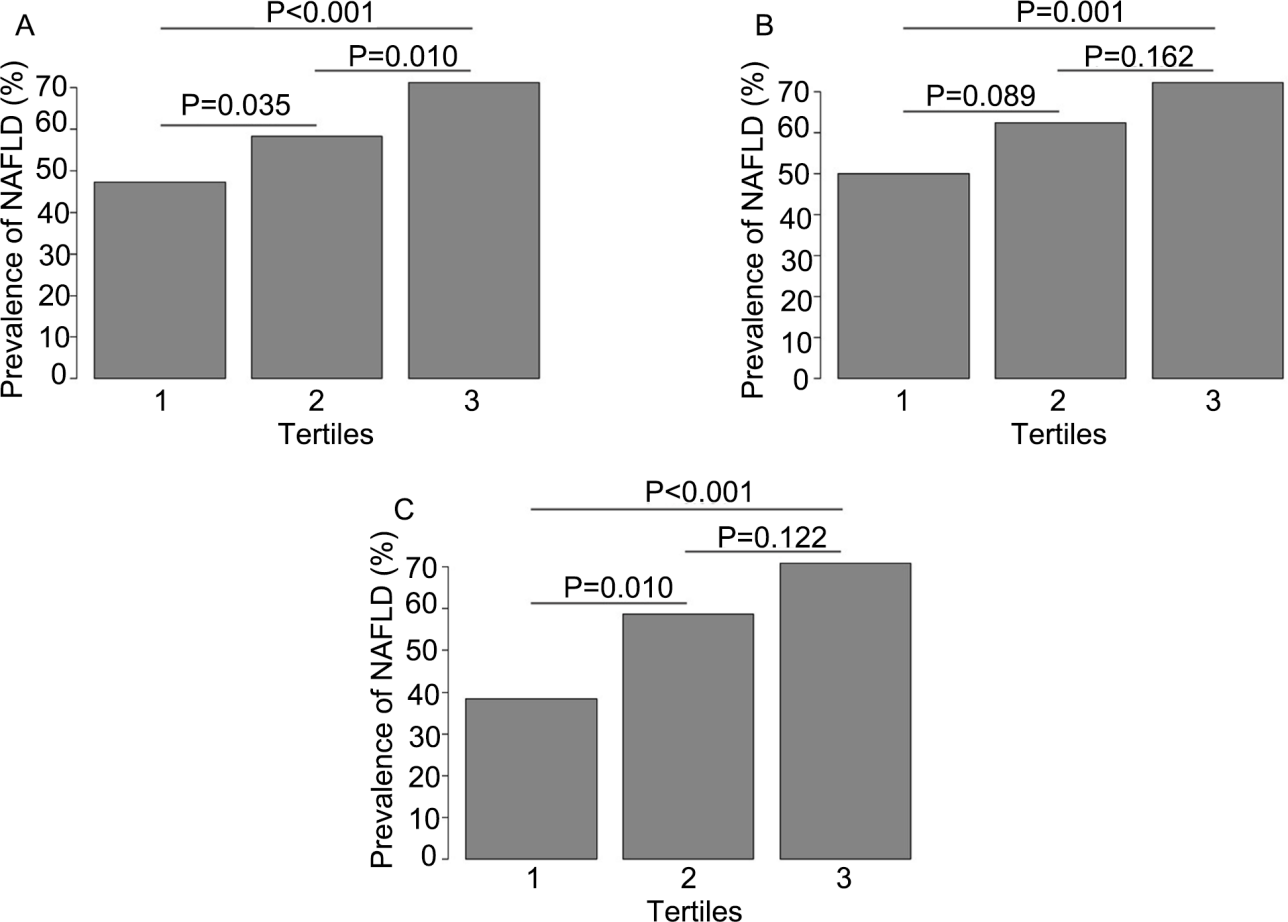
Prevalence of nonalcoholic liver disease (NAFLD) according to tertiles of serum uric acid (SUA). (A) All participants with type-2 diabetes mellitus (T2DM). SUA tertile 1, <280 µmol/L; SUA tertile 2, 280–349 µmol/L; SUA tertile 3, >349 µmol/L. * P* for trend <0.001. (B) Men with T2DM. SUA tertile 1, <300 µmol/L; SUA tertile 2, 300–361 µmol/L; SUA tertile 3, >361 µmol/L; * P* for trend <0.001. (C) Women with T2DM. SUA tertile 1, <260 µmol/L; SUA tertile 2, 260–328 µmol/L; SUA tertile 3, >328 µmol/L; *P* for trend <0.001.

### Associations of SUA with other clinical parameters

Pearson correlation analyses (Table 3) demonstrated that in both genders, SUA level was significantly positively correlated with BMI (*P* < 0.001 for both genders), TG (*P* < 0.001 for both genders), ALT (*P* < 0.001 for men and *P* = 0.006 for women), AST (*P* = 0.049 for men and *P* < 0.001 for women) and HOMA-IR (*P* = 0.015 for men and *P* = 0.034 for women) and negatively correlated with eGFR (*P* = 0.035 for men and *P* < 0.001 for women). SUA was also negatively correlated with HDL-C in women (*P* = 0.010) but not men. SUA was not correlated with age, duration of T2DM, SBP, DBP, TC, LDL-C, FPG or HbA1c (Table 3). Multiple linear regression analysis (using the stepwise method) revealed that BMI (positively), TG (positively) and eGFR (negatively) were independently related to SUA in both men and women with T2DM (*P* < 0.01; Table 3). Multiple linear regression also indicated that SUA level was positively associated with AST (*P* < 0.001) and ALT (*P* = 0.046) in women (Table 3).

### Prevalence of NAFLD according to tertiles of SUA in patients with T2DM

Figure 1 displays data for the prevalence of NAFLD according to tertiles of SUA. When all study participants were analyzed together, the prevalence of NAFLD increased progressively from the lowest SUA tertile to the highest SUA tertile (47.2%, 58.3% and 71.1%; *P* for trend < 0.001; Fig. 1A). Progressive increases in NAFLD prevalence with increasing SUA tertile (from lower to upper) were also observed in men (50.0%, 62.4% and 72.2%; *P* for trend < 0.001; Fig. 1B) and women (38.5%, 58.7% and 70.8%; *P* for trend < 0.001; Fig. 1C). However, when compared with tertile 1, a significant rise in NAFLD prevalence was observed for both tertile 2 (*P* = 0.010) and tertile 3 (*P* < 0.001) in women but only tertile 3 (*P* = 0.001) in men.

Logistic regression analysis (using SUA tertile 1 as the reference group) after adjustment for age, duration of T2DM, BMI, SBP, alcohol intake and eGFR revealed that the odds of NAFLD were significantly increased for tertile 2 and tertile 3 in all patients and in women but only for tertile 3 in men (see Table 4 for details). After further adjustment for TG, HbA1c, HOMA-IR, statin use, anti-hypertensive drug use and history of hyperuricemia, SUA tertiles were not associated with NAFLD in men, whereas the odds of NAFLD remained significantly elevated for tertile 3 in women (Table 4). A similar result was obtained when the model was further adjusted for C-peptide, HDL-C and, for females only, ALT and AST, i.e., the odds of NAFLD were significantly elevated only for tertile 3 in women (Table 4). However, there appeared to be trends toward increased odds of NAFLD for tertile 2 in women (*P* = 0.122) and tertiles 2 and 3 in men (*P* = 0.074 and 0.118, respectively).

**Table 4:** Logistic regression analysis of the association between tertiles of serum uric acid and prevalence of nonalcoholic fatty liver disease with type-2 diabetes mellitus.

	Tertile 1	Tertile 2	Tertile 3	*P value for trend*
Model 1				
All	1	1.769 (1.142–2.741)	2.368 (1.485–3.776)	
*P*		0.011	<0.001	<0.001
Men	1	1.595 (0.865–2.943)	2.120 (1.114–4.037)	
*P*		0.135	0.022	0.021
Women	1	2.231 (1.175–4.234)	3.696 (1.798–7.598)	
*P*		0.014	<0.001	<0.001
Model 2				
All	1	1.702 (1.067–2.715)	2.059 (1.246–3.400)	
*P*		0.026	0.005	0.004
Men	1	1.862 (0.963–3.602)	1.782 (0.880–3.607)	
*P*		0.065	0.108	0.093
Women	1	1.819 (0.913–3.625)	2.392 (1.101–5.200)	
*P*		0.089	0.028	0.025
Model 3				
All	1	1.651 (1.030–2.647)	2.096 (1.262–3.481)	
*P*		0.037	0.004	0.004
Men	1	1.849 (0.943–3.627)	1.768 (0.866–3.608)	
*P*		0.074	0.118	0.102
Women	1	1.747 (0.861–3.543)	2.336 (1.041–5.242)	
*P*		0.122	0.040	0.036

**Notes.**

Data are presented as o0dds ratio (95% confidence interval) compared with tertile 1. Participants without nonalcoholic fatty liver disease (NAFLD) were defined as 0 and those with NAFLD as 1.

Model 1 adjusted for age, duration of diabetes mellitus, body mass index, systolic blood pressure, alcohol intake and estimated glomerular filtration rate Model 2 model 1 with additional adjustment for triglycerides, glycated hemoglobin, homeostasis model assessment of insulin resistance, statin use, anti-hypertensive drug use and history of hyperuricemia Model 3 model 2 with additional adjustment for C-peptide, high-density lipoprotein-C and, for females only, alanine aminotransferase and aspartate aminotransferase. SUA tertile 1 <280 µmol/L for all patients, <300 µmol/L for men and <260 µmol/L for women SUA tertile 2 280–349 µmol/L for all patients, 300–361 µmol/L for men and 260–328 µmol/L for women SUA tertile 3 >349 µmol/L for all patients, >361 µmol/L for men and >328 µmol/L for women

## Discussion

An important finding of this cross-sectional study of patients with T2DM in the community was that SUA was higher in the NAFLD group than in the non-NAFLD group for both women and men. Furthermore, SUA correlated positively with BMI and TG and negatively with eGFR in both genders. Logistic regression analysis with adjustment for age, duration of T2DM, BMI, SBP, alcohol intake and eGFR showed that SUA tertiles 2 and 3 in women and SUA tertile 3 in men were associated with significantly increased odds of NAFLD (as compared with SUA tertile 1), with a trend toward increased odds for tertile 2 in men. After rigorous adjustment for multiple clinical parameters, a significant increase in NAFLD prevalence was only observed for SUA tertile 3 in women. However, there were clear trends suggesting an increased prevalence of NAFLD for tertile 2 in women (*P* = 0.122) and tertiles 2 and 3 in men (*P* = 0.074 and 0.118, respectively) as compared with tertile 1. Taken together, our data suggest that although there seems to be an association between SUA and NAFLD that is stronger in women than in men, this association may largely be mediated by other metabolic factors.

NAFLD is the most common cause of chronic liver disease globally, and the positive association between NAFLD and T2DM is well established (Kitade et al., 2017; Mikolasevic et al., 2016; Neuschwander-Tetri, 2017). The prevalence of NAFLD in the 597 patients with T2DM in our study was 59.0%, consistent with a previous meta-analysis of 24 studies that yielded a pooled prevalence of 59.7% (95%CI [54.3–64.9]%) (Dai et al., 2017). This illustrates the high prevalence of NAFLD in patients with T2DM, highlighting the importance of identifying factors predictive of incidental NAFLD.

Many clinical investigations have reported that an elevated level of SUA is associated with a higher prevalence of NAFLD (Cai et al., 2014; Cai et al., 2013; Lee et al., 2010a; Lee et al., 2010b; Li et al., 2009; Lin et al., 2015; Liu et al., 2016; Moon, 2013; Shih et al., 2015; Sirota et al., 2013; Yamada et al., 2010). Moreover, an increased SUA level is related to a higher risk of new-onset NAFLD (Bai et al., 2018; Zhou, Wei & Fan, 2016) and more severe NAFLD (Liu et al., 2017; Petta et al., 2011; Sirota et al., 2013). This previous research not only suggests that measurement of SUA could be used to predict the presence or future occurrence of NAFLD but also implicates SUA as possibly contributing to the development of NAFLD. Some preclinical studies have suggested that uric acid might directly influence fat accumulation and hepatic steatosis by inhibiting insulin signaling to cause insulin resistance (Zhu et al., 2014), inducing mitochondrial oxidative stress (Lanaspa et al., 2012) or generating endoplasmic reticulum stress (Choi et al., 2014). It has even been proposed that suppression of SUA levels might be a potential new therapy for NAFLD (Sun et al., 2016). Nonetheless, the mechanisms linking elevated SUA levels and NAFLD have not yet been fully characterized in the clinical setting, and it is possible that the effects of SUA are not direct but are mediated via other risk factors for NAFLD. In this study, Pearson correlation analysis revealed that SUA level was positively correlated with BMI, TG and HOMA-IR and negatively correlated with eGFR in both men and women. Furthermore, SUA was also negatively correlated with HDL-C in women, with a trend toward a negative correlation in men (*P* = 0.082). Our findings are broadly consistent with a previous study of patients with T2DM in China, which also suggested that SUA was positively correlated with BMI and TG and negatively correlated with eGFR and HDL-C (Fan et al., 2016). Higher BMI, elevated TG and impaired insulin resistance are factors known to be associated with an increased risk of NAFLD (Kitade et al., 2017; Mikolasevic et al., 2016; Neuschwander-Tetri, 2017), raising the possibility that the relation between SUA and NAFLD is mediated, at least in part, by these other factors.

Comparisons of NAFLD prevalence between SUA tertiles suggested that the association between elevated SUA level and increased prevalence of NAFLD was stronger in women with T2DM than in men with T2DM (Fig. 1), and this possibility was supported by the results of the univariate analysis (Table 1). Previous studies of general populations have reported conflicting results regarding sex differences in the association between SUA and NAFLD. A large-scale study of people attending medical centers in China found that the association between elevated SUA and increased prevalence of NAFLD was greater in females than in males (Wu et al., 2015), in broad agreement with our findings. However, another study in China concluded that the relationship between elevated SUA and increased prevalence of NAFLD was stronger in males than in females (Yu et al., 2017), which would not be consistent with our current data. Furthermore, a previous investigation of patients with T2DM in China determined that the association between elevated SUA and increased prevalence of NAFLD was stronger in men than in women (Fan et al., 2016). In fact, this latter study found no significant differences in NAFLD prevalence between tertiles of SUA in women (despite a trend toward increasing NAFLD prevalence with increasing SUA tertiles), whereas significant differences were detected for men (Fan et al., 2016). Furthermore, univariate logistic regression analysis revealed a significant association between increasing SUA tertile and higher NAFLD prevalence for men but not for women (Fan et al., 2016). Interestingly, after adjustment for age, duration of T2DM, SBP, DBP and BMI, SUA tertiles showed significant associations with NAFLD in men but not women, and this gender difference was maintained after further adjustment for T2DM therapy, FPG, HbA1C, TG, TC, LDL-C, HDL-C, eGFR and HOMA-IR (Fan et al., 2016). By contrast, the present study found no significant independent associations of SUA tertiles with NAFLD in men after adjustment for age, duration of T2DM, BMI, SBP, alcohol intake, eGFR, TG, HbA1c, HOMA-IR, statin use, anti-hypertensive drug use and history of hyperuricemia, while a significant association in women was observed only for tertile 3 (i.e., increased odds of NAFLD in tertile 3 vs. tertile 1). Since both our study and that of Fan et al. (2016) used comparable criteria to enroll Chinese patients with T2DM, it is perhaps unlikely that the differences in results were due to notable dissimilarities in population characteristics. Instead, the apparent discrepancies between our results and those of Fan et al. (2016) may reflect differences in the analytical methods used. For example, Fan et al. (2016) adjusted for fewer factors in the multivariate analysis and defined the SUA tertiles using different ranges (SUA tertiles 1, 2 and 3 were defined as ≤265.6, 265.6–338.4 and >338.4 µmol/L for men and ≤232.0, 232.0–299.4 and >299.4 µmol/L for women). An additional potential limitation of the study by Fan et al. (2016) is that they did not adjust for ALT and AST. Elevated levels of liver enzymes are known to be associated with an increased presence and severity of NAFLD (Bi et al., 2014; Chien-Min & Cheng-Chuan, 2017; Tasneem, Luck & Majid, 2018). Furthermore, a study of patients with T2DM found that those with NAFLD had significantly higher levels of ALT, AST and GGT (consistent with our data), and SUA concentration was positively correlated with the levels of all three liver enzymes (Chien-Min & Cheng-Chuan, 2017). Therefore, it is also possible that the levels of these liver enzymes may mediate part of any association between SUA tertiles and NAFLD. However, in our analysis, additional adjustment for AST and ALT did not alter the finding that tertile 3 was associated with significantly increased odds of NAFLD (vs. tertile 1) in women.

Overall, our findings suggest that the association between elevated SUA and increased prevalence of NAFLD is at best weak after rigorous adjustment for relevant metabolic factors. Nonetheless, it should be noted that we did observe a clear trend toward an association between higher SUA tertile and higher prevalence of NAFLD in both men and women even after adjustment for numerous metabolic factors. Thus, it cannot be completely excluded that our study was underpowered to detect real independent associations of SUA tertiles with NAFLD prevalence.

Previous research has indicated that hyperuricemia is common in patients with T2DM. For example, the prevalence of hyperuricemia in patients with T2DM was reported to be 45.5% in Indonesia (Sunjaya & Sunjaya, 2018) and 32.6% in China (Wang et al., 2013). This contrasts with our finding that the prevalence of hyperuricemia was only 1.5% (9 of 597 patients). The likely reason for this apparent discrepancy is that our study did not enroll patients who were taking medications that might affect SUA, and this would have excluded patients with hyperuricemia who were receiving therapy with uric acid-lowering drugs.

This study has some limitations. First, this was a single-center study so the generalizability of the findings remains unknown. Second, the sample size was small; hence it is possible that the study was underpowered to detect some real differences between groups. Third, this was a cross-sectional study, preventing any conclusions being drawn regarding cause and effect. Fourth, the diagnosis of NAFLD was made using ultrasonography, but this technique may miss cases of mild liver steatosis detectable by liver biopsy. Fifth, due to the diagnostic method used, it was not possible to examine the relationship between SUA and NAFLD severity.

## Conclusion

Our study provides evidence of a positive association between SUA level and NAFLD prevalence that may be stronger in women than in men. This association may be largely mediated by other metabolic factors since SUA tertiles were not independently associated with NAFLD in multivariate analyses, except for tertile 3 in women, after adjustment for these metabolic factors. Nonetheless, considering the trends toward associations for tertiles 2 and 3 in men and tertile 2 in women, it is possible that our study was underpowered to detect a real independent association between elevated SUA level and increased NAFLD prevalence.

##  Supplemental Information

10.7717/peerj.7563/supp-1 Data S1 Raw data

## Additional Information and Declarations

### Data Availability

The following information was supplied regarding data availability: Raw data is available as a Supplemental File.
